# Intramolecular Hydrogen Bonding Based CEST MRI Contrast Agents As an Emerging Design Strategy: A Mini-Review

**DOI:** 10.1021/acsomega.4c02296

**Published:** 2024-06-17

**Authors:** Zinia Mohanta, Sadakatali Gori, Michael T. McMahon

**Affiliations:** † Russell H. Morgan Department of Radiology and Radiological Science, Johns Hopkins University School of Medicine, Baltimore, Maryland 21205, United States; ‡ F.M. Kirby Research Center for Functional Brain Imaging, Kennedy Krieger Research Institute, Baltimore, Maryland 21205, United States; § Center for Translational Pharmacology, Department of Pharmacy and Pharmaceutical Sciences, St. Jude Children’s Research Hospital, Memphis, Tennessee 38105-3678, United States

## Abstract

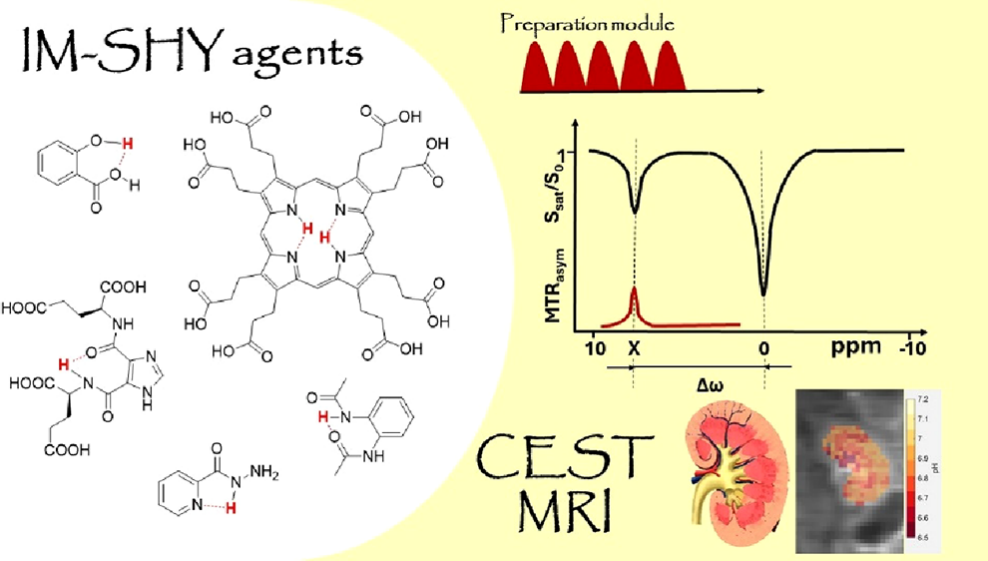

Intramolecular hydrogen bonding-based chemical exchange saturation transfer magnetic resonance imaging (CEST MRI) contrast agents represent an innovative design strategy aiming to overcome limitations in diamagnetic CEST (diaCEST) MRI contrast agent specificity and also those associated with traditional metal-based MRI contrast agents. Ward and Balaban’s proposal of small diamagnetic compounds marked a paradigm shift in contrast-based radiologic research, inspiring extensive investigations since 2000. These contrast agents leverage labile hydrogen bonds, serving as chemical exchange sites to induce saturation of water. The selective manipulation of radiofrequency (RF) allows for optimized signal contrast in soft tissue, with a significant signal amplification even at low probe concentrations, mitigating concerns about dose-dependent toxicities. This mini-review delves into the evolution of CEST MRI, its classification, and the strategic design principles of synthetic small molecules containing intramolecular hydrogen bonds. With a focus on applications and potential clinical relevance, the authors highlight the promising role of intramolecular hydrogen bonding-based CEST MRI in diverse medical contexts, especially renal imaging and pH mapping, paving the way for enhanced molecular imaging capabilities. Ongoing research endeavors aim to further optimize and expand the utility of these contrast agents, underscoring their transformative potential in clinical diagnostics and imaging.

## Introduction

Magnetic resonance imaging (MRI) is an established imaging modality that provides outstanding capabilities for detecting soft tissue abnormalities. MRI contrast agents are used to create a visible change in water signal intensities typically by altering the longitudinal or transverse relaxation times of water within soft tissue. This results in highlighting the region where the contrast agent accumulates, which is very helpful to detect pathology. In fact, approximately 33% of all MRI scans involve injection of contrast.^1^ The current armamentarium of MRI contrast agents consists of paramagnetic complexes containing gadolinium, manganese and superparamagnetic nanoparticles of iron oxides, etc.^2^ Gadolinium based contrast agents are widely used in the clinic, with approximately 59 million administrations per year;^1^ however, caution is urged for patients with severe renal impairment due to its toxicity. Furthermore, widespread usage of gadolinium has raised additional concerns due to its accumulation in water systems after patients urinate the gadolinium contrast.^3^ As a result of these issues, new alternatives are needed.

**Figure 1:**
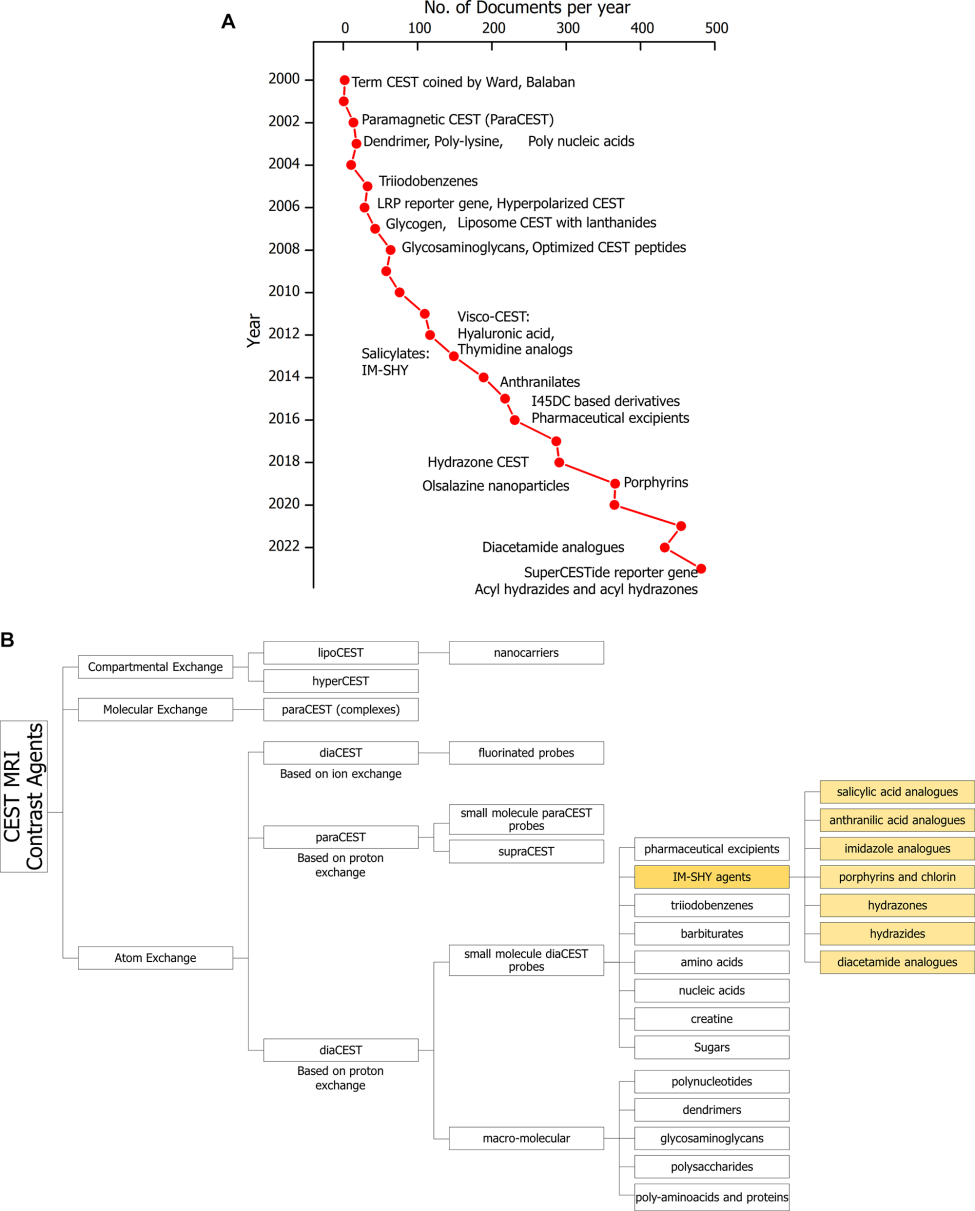
An overview and classification of the evolution of CEST MRI contrast agents. (A) The graph shows the trend of increase in number of articles per year and highlights advances in CEST MRI agents. Articles include research articles, reviews, book chapters, conference papers and editorials. Source: Scopus (Search Strategy: cest AND mri, search within all fields, limited to Document type: Article, Review, Book Chapter, Conference Paper, Book). (B) Classification of CEST MRI contrast agents and types of Intramolecular hydrogen bonding (IM-SHY) based contrast agents.

Wolff and Balaban proposed use of saturation transfer to selectively highlight compounds with labile protons.^4^ Using this technique, molecules with labile hydrogens can serve as chemical exchange shunts to transfer saturation to the surrounding water. Controlling the rate of this signal loss transfer via manipulation of radiofrequency (RF) pulses allows optimization and enhancement of signal contrast in soft tissue. As the labile protons on small molecules serve as conduits for saturation transfer to surrounding water molecules, the signal can be amplified multifold and even high micromolar to low millimolar concentrations of the labile protons on such probes is sufficient to obtain high spatial resolution MR images. This underlying principle, termed chemical exchange saturation transfer (CEST) inspired a paradigm shift in contrast-based radiologic research and has been widely investigated since its introduction. In another seminal work, Ward and Balaban identified 32 diamagnetic compounds which displayed CEST contrast (diaCEST agents) including sugars, nucleosides, barbituric acid derivatives, imino acids, and imidazole derivatives.^5^ Soon after, Aime, Sherry, and colleagues discovered paramagnetic complexes which displayed contrast through proton exchange with water^6^ (paraCEST agents). Figure 1a depicts the evolution CEST MRI contrast agents since the inception of this concept. Several reviews and one textbook have attempted to compile and classify the growing categories of novel CEST probes.^7,8^Figure 1b displays a breakdown of the types of CEST MRI contrast agents emphasizing the growing array of intramolecular bond shifted hydrogens (IM-SHY) CEST MRI contrast agents, which are the focus of this review.

While naturally occurring diaCEST probes can possess excellent biocompatibility, synthetic small molecule probes can be further optimized by tuning their exchange properties and response of these to environmental factors for specific medical applications. The contrast efficiency of a molecular probe is dependent on a number of factors such as exchange rate (*k*_ex_), labile proton resonance frequency with respect to bulk water (Δω), pH, and concentration of the probe in targeted tissue. The design of new probes is primarily focused on developing molecules with a large offset from the water signal to selectively irradiate the probe H-bond and to afford a larger exchange rate; and therefore, better contrast efficiency. Initially barbituric acid analogs, nucleic acids, and triodobenzenes were identified with improved CEST characteristics.^5,9^ However, with the ideal CEST properties established to be Δω > 5, *k*_ex_ ∼ 1000 s^–1^ based on the saturation pulse trains achievable using a standard body coil on clinical 3 T scanners,^8,10^ a more aggressive search has commenced to find molecules which possess protons with these characteristics. These highly shifted diaCEST scaffolds are the subject of this review, with the highly shifted labile protons realized through inclusion of appropriate strength intramolecular H-bonding (downfield shift) or through diatropic ring currents in π-electron-rich aromatic compounds (upfield shift). A number of the molecules have been extensively studied as therapeutics and therefore should be very biocompatible. In particular, we have focused this review on small molecules as these may enable higher spatial resolution and more specific detection on clinical 3 T scanners.

## Classification of CEST MRI Contrast Agents

Several classes of CEST agents with strong image contrast have been reported in the past two decades. There are three primary categories based on the type of chemical exchange underlying the CEST technology; atom (proton) exchange, molecular exchange, and compartmental exchange.^11^ Atom exchange CEST probes contain exchangeable, labile atoms (primarily a proton) such as in hydroxy, amide, or amine protons. This proton(s) may be tethered to a small molecule scaffold as in case of a diaCEST agent, to a polymeric scaffold with a centrally chelated metal core as in case of a paraCEST agent, or to a macro-/supra-molecular host scaffold at one of the functional group moieties. Molecular exchange CEST probes contain exchangeable molecules (e.g., water molecule) which are shifted by a metal center. In such probes, the molecular exchange rate is fairly slow compared to MRI agents producing T_1_ contrast, enabling a detectable shift when bound to the complex. Thus, in the case of a coordinated water molecule, proton exchange is indistinguishable from the molecular exchange. Compartmental exchange probes constitute compartmentalized water molecules or hyperpolarized xenon that exchange between two compartments, with the presence of shift agents or the interactions of xenon with a cryptophane cage inducing a shift when water or xenon is inside the compartment.^12^ Because of this, and slow exchange between compartments, inner molecules can be saturated and this signal loss transferred to the exterior water of xenon creating image contrast. The above-mentioned classes are further divided into subcategories as depicted in Figure 1. In this review we will focus on intramolecular hydrogen bonding based agents.

## Intramolecular Hydrogen Bonding Based CEST MRI Contrast Agents

**Figure 2:**
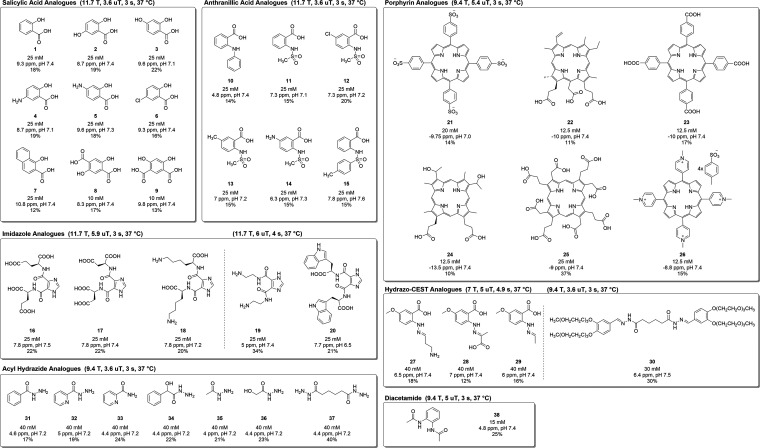
Structures of intramolecular H-bonded CEST-MRI agents described previously. IM-SHY probes that demonstrated more than 3.5 ppm shift in signal from water and showed more than 10% contrast enhancement are categorized here. General experimental conditions are noted with each probe category. For each probe, concentration, chemical shift, % contrast enhancement, and exchange rate are noted under the structures.

The intramolecular hydrogen bonding-based IM-SHY agents are a class of diaCEST contrast agents which involve molecules having intramolecular hydrogen bonds and leveraging the slowed chemical exchange between hydrogen atoms engaged in such bonding and free water protons for tuning CEST properties. Figure 2 further categorizes IM-SHY probes according to their respective molecular scaffolds; viz., salicylic acid, anthranilic acid, imidazole, acyl hydrazide, hydrazo-CEST, as well as a diacetamide probe. General experimental conditions for each category are noted over the respective set of probes and concentration, chemical shift, % contrast enhancement, and *k*_ex_’s are noted for individual probes.

## Factors Affecting CEST Contrast Enhancement

### Experimental Parameters Affecting CEST Properties and Contrast Enhancement

#### Magnetic Field Strength

CEST contrast increases with *B*_0_.^13^ The enhancement in CEST contrast at higher magnetic fields is due in part to longer T1w, enabling extended saturation in the water pool. Other benefits include reduced water saturation interference due to the better separation of the signals from water which increases proportionally to field strength.^11^

#### Saturation Time

Saturation time is kept long (seconds) to maintain labeling efficiency.^14^

#### Saturation Power

The saturation process is nonlinear and increasing the saturation power may not increase CEST contrast once the exchangeable pool is fully saturated.^15^ In fact, this can reduce contrast due to increasing the direct saturation of water by the saturation pulse.

#### B_0_ Inhomogeneity

B_0_ inhomogeneity causes artifacts in CEST contrast maps.^13^

### Properties of Molecules Affecting CEST Properties and Contrast Enhancement

#### Exchange Rate and Labile Proton Chemical Shift

To exhibit CEST MRI contrast, the exchange rate (*k*_ex_) is preferably less than the difference in chemical shift between the exchangeable protons and the water protons (Δω): Δω ≥ *k*_ex_(8)

#### Concentration of the Agent

CEST contrast depends on agent concentration.^16^ If the concentration is extremely low, then it is difficult to detect any signal and if it is too high, then there is stronger back exchange which reduces the contrast. Optimization of concentration becomes important. Ratiometric CEST MRI allows calculation of CEST effect from multiple labile protons of same molecule and is independent of concentration of the agent.

#### Saturation Efficiency

The saturation efficiency of rapidly exchanging protons can be approximated using the equation,
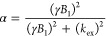
where γ is the gyromagnetic ratio, *B*_1_ is the magnetic field strength, and *k*_ex_ is the exchange rate. In this context, higher RF power is required for effective saturation, which can be problematic in vivo due to specific absorption rate (SAR) limits.^11^

#### pH

The chemical exchange rate of a CEST agent varies as a function of pH and thus the CEST contrast also varies systematically along a range of pH values. Suitable CEST agents for in vivo applications must possess reasonable exchange properties around neutral pH.

#### Salicylic Acid Analogues

Salicylic acid (**1**) is the primary metabolite of aspirin and contains labile protons that resonate 9.3 ppm from water. Yang et al. investigated the CEST properties of salicylic acid and its analogues.^17^ In vitro measurements revealed that salicylic acid exhibited pH-dependent proton exchange rates, with a near ideal *k*_ex_ ∼ 500 s^–1^ in phosphate buffered saline (PBS) at neutral pH values. The study explored eight analogues of salicylic acid (**1**–**9**), demonstrating how electronic modifications to the phenol ring affected CEST contrast. The analogues exhibited varying chemical shift values up to 10.3 ppm, with the meta position −OH (**2**) or −NH_2_ (**4**) group showing a reduction in chemical shift. Notably, the introduction of a naphthalene ring in 1-hydroxy-2-naphthoic acid (**7**) shifted the CEST peak signal further downfield. To assess in vivo detectability, salicylic acid (**1**) was injected into mice, resulting in a pronounced increase in CEST contrast in the kidneys, peaking 7 min postinjection. The large shift of salicylic acid (**1**) at 9.3 ppm distinguished it from common metabolite signals. The authors observed a CEST contrast of 6.0 ± 0.8% in the kidneys. Salicylic acid and its analogs (**1**–**9**) represent a promising set of diaCEST probes, offering low toxicity and potential improvements in sensitivity for existing CEST methods. In a separate study, 44 hydrogen-bonded phenols were investigated for their potential as CEST MRI contrast agents to characterize the stereoelectronic effects of a number of substituents on the CEST properties.^10^ The authors demonstrated that the exchangeable protons in phenols could be finely tuned through ring substitution, enabling adjustment of the proton exchange rate and chemical shift to maximize CEST contrast. Salicylic acid (**1**) served as a reference, and the study explored substitutions at the 3-, 4-, 5- and 6-positions on 2-hydroxybenzoic acids and demonstrated that subtle modifications could dramatically alter hydrogen bonding, chemical shift, and exchange rates. Additionally, compounds with multiple IM-SHY cores, like 2,5-dihydroxyterephthalic acid (**8**) and 4,6-dihydroxyisophthalic acid (**9**), exhibited high sensitivity as diaCEST probes. The study also revealed that substitutions at the 4 and 5 positions were fairly benign for impacting CEST properties, enabling facile attachment of this high performance CEST moiety to polymers. A number of these probes are very well tolerated, with the additional benefit that the large chemical shift of these probes can be calculated using quantum chemistry.^18^ Cumulatively, these observations were encouraging for enabling 3 T detection in a variety of medical imaging studies.

**Figure 3:**
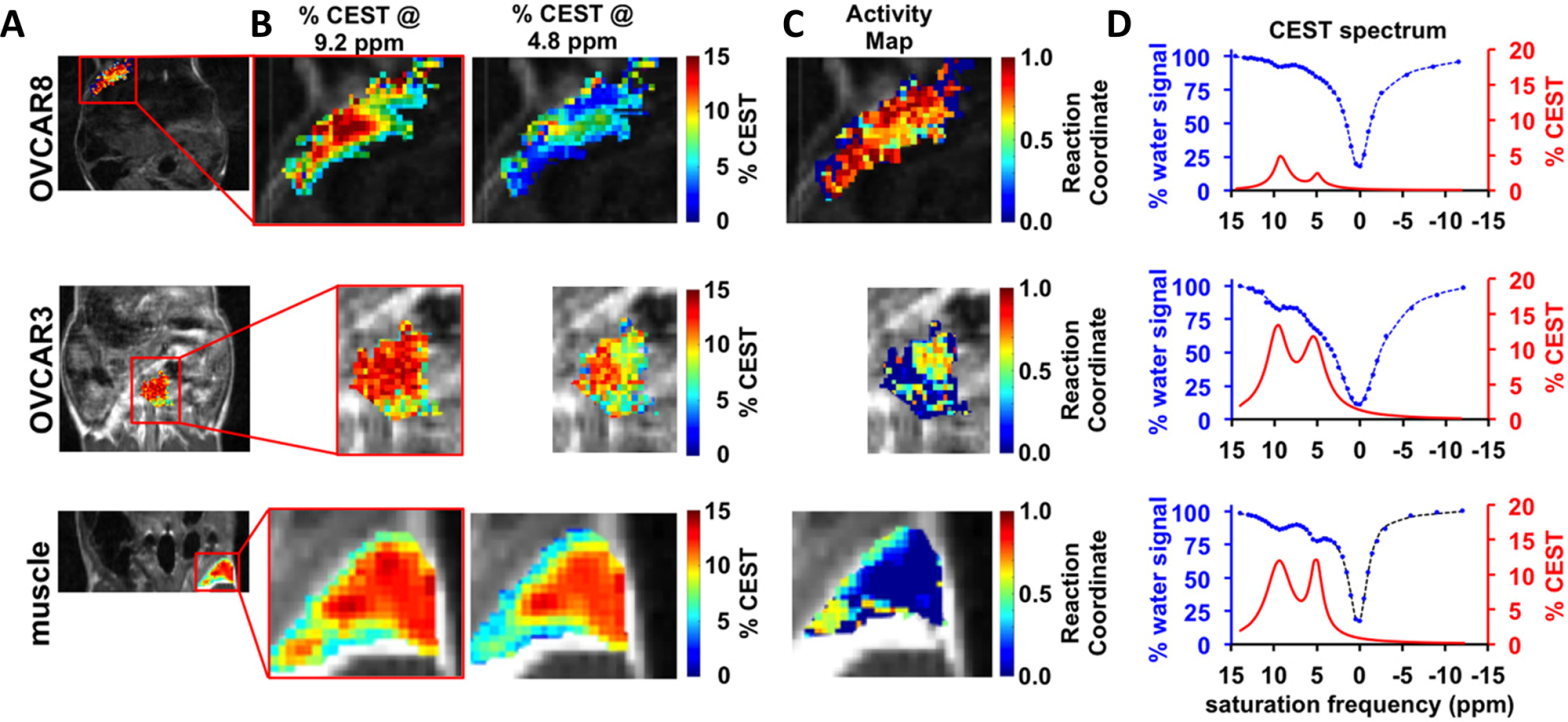
In vivo catalyCEST MRI. (A) Anatomical images reveal the regions studied in OVCAR-8 tumor, OVCAR-3 tumor, and muscle tissue, with the red rectangle indicating focus areas. (B) Parametric maps at 9.2 and 4.8 ppm display effective detection of CEST signals in both tumor and muscle tissues. (C) GGT enzyme activity maps exhibit high activity in OVCAR-8 tumor, low activity in OVCAR-3 tumor, and no activity in muscle tissue. (D) CEST spectra (blue) and signals (red) in the tumor and muscle regions demonstrate the agent’s detection sensitivity and relative signal ratios. (Adapted with permission from Ref (24), Copyright John Wiley and Sons.)

Salicylates have now been utilized in a variety of in vivo applications. Song et al. have employed unmodified salicylates for assessment of brain perfusion territory after blood brain barrier opening.^19^ Pavuluri et al. employed injectable aspirin to visualize salicylic acid uptake in orthotopic breast tumor models.^20^ The first prototype of polymeric salicylate probes was prepared and tested by Lesniak et al. whereby salicylate was attached to a dendrimer for visualizing convection enhanced delivery to brain tumors.^21^ Pagel, Sinharay, Bulte and colleagues have employed the salicylic acid moiety to tumor enzyme activatable probes including for detecting cathepsin B activity,^22^ alkaline phosphatase activity,^23^ γ-glutamyl transferase activity,^24^ furin activity,^25^ and others,^26^ An example of this is shown in Figure 3, where a γ-glutamyl transferase cleavable probe was developed by Sinharay et al. As is shown, maps of γ-glutamyl transferase activity were produced after intratumoral administration of probe. In another exemplary study, salicylate was conjugated to the poly(isobutylene–maleic anhydride) scaffold along with the l-lysine urea-l-glutamate (KUE) targeting group to detect the presence of the integral membrane protein prostate specific membrane antigen (PSMA), an important target for imaging and therapy of castration-resistant prostate cancer.^27^ In summary, the high specificity and sensitivity of salicylate probes has been realized for a variety of in vivo medical imaging applications.

#### Anthranilic Acid Analogues

Song et al. conducted a study of the CEST properties of various anthranilic acid analogues. It was observed that while anthranilic acid, an −NH analogue of salicylic acid, did not yield any CEST contrast, a number of *N*-alkyl, *N*-aryl, *N*-acyl, and *N*-sulfonyl substitutions resulted in strong contrast for this scaffold.^28^ Anthranilates rely on the exchange of N–H protons, and in the case of *N*-phenyl anthranilic (**10**), the labile N–H resonated at 4.8 ppm and exhibited a broader peak in the CEST spectrum, indicating faster exchange. The *N*-acyl analogue (not presented in this review) had a frequency offset of 9.8 ppm but displayed lower CEST enhancement compared to *N*-aryl analogues. *N*-Sulfonyl analogues (**11**–**15**) resonated between 6.3 to 7.8 ppm. One specific analogue, 5-chloro-2-[(methylsulfonyl)amino] benzoic acid (**12**), a *N*-sulfonyl analogue, proved to be highly sensitive, displaying a 2–3% signal enhancement during in vivo CEST imaging. At this stage, these probes have largely been tested in solution, however it is expected that they would work well for in vivo applications.

#### Imidazole Analogues

Yang et al. investigated a number of imidazoles with heterocyclic N–H in 2016 to elicit CEST contrast and identified derivatives with intramolecular hydrogen bonding with excellent CEST properties.^29^ Imidazole-4,5-dicarboxamide (I45DC) showed poor saturation transfer efficiency due to its fast exchange rate with water. Modification to 4,5-bis[(Glu)carbonyl]-1H-imidazole, also called I45DC diGlutamate (diGlu) (**16**), resulted in significant contrast at 7.8 ppm downfield from water at neutral pH. I45DC-diGlu (**16**) was also found to produce good pH-dependent contrast for measurements. Additional water-soluble analogues 4,5-bis[(Asp)carbonyl]-1H-imidazole (**17**) and 4,5-bis[(Lys)carbonyl]-1H-imidazole (**18**) were synthesized. Both analogues demonstrated CEST contrast, with compound 4,5-bis[(Asp)carbonyl]-1H-imidazole (**17**) having a slightly slower exchange rate than I45DC-diGlu (**16**). Despite some limitations, such as suboptimal exchange rates for certain scanners, the imidazole-based agents are considered promising alternatives for pH imaging, offering tunability through modification and potential applications in medical diagnostics.

**Figure 4:**
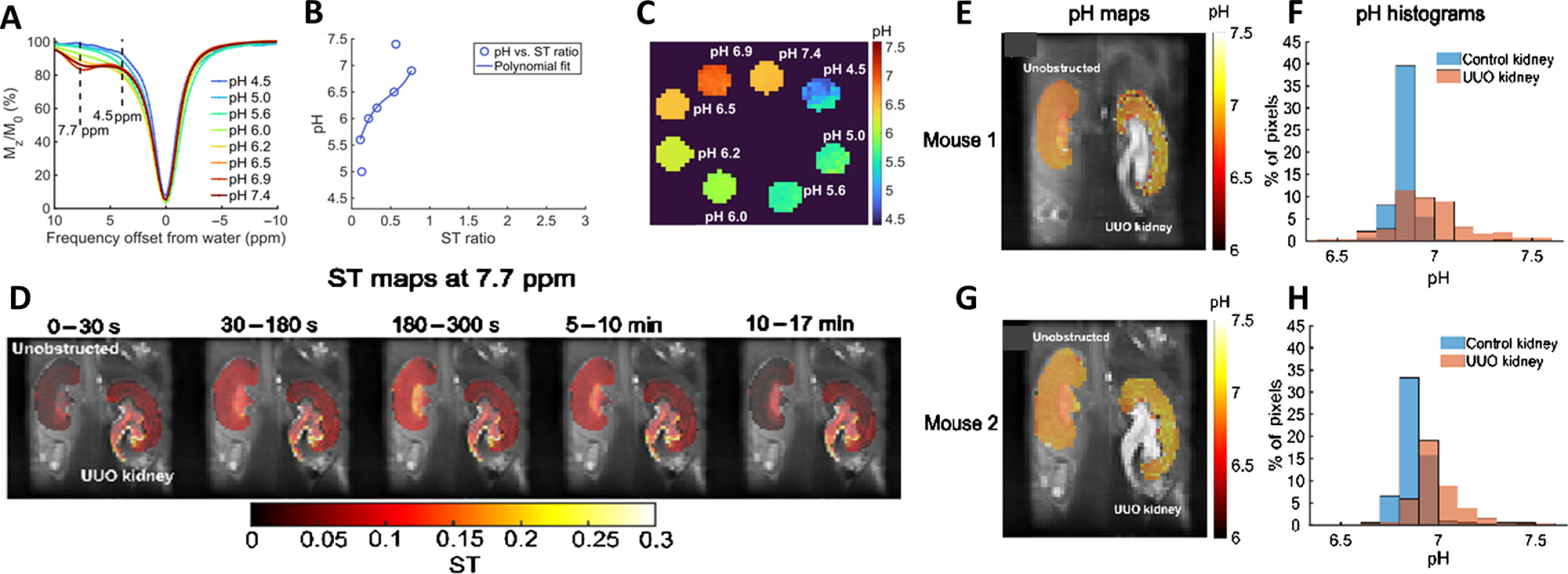
In vitro and in vivo CEST MRI for I45DC-diGlu. (A) In vitro CEST Z-spectra acquired for I45DC-diGlu (**16**) at a concentration of 25 mM, *t*_sat_ = 4 s and temperature maintained at 37 °C. (B) ST_ratio_ plotted against pH, and (C) pH maps corresponding to I45DC-diGlu (**16**). (D) In vivo CEST MRI of unilateral ureter obstruction (UUO) mice injected with I45DC-diGlu (**16**). Saturation transfer (ST) maps at 7.7 ppm for a representative mouse, generated by averaging images acquired at different time intervals postadministration of I45DC-diGlu (**16**), superimposed on high-resolution T2-weighted anatomical images. (E and G) Extracellular pH (pHe) maps, and (F and H) pH histograms for two UUO mice post I45DC-diGlu (**16**) injection. (Adapted with permission from Ref (30), John Wiley and Sons.)

Bo and colleagues further developed the I45DC scaffold via synthesis and evaluation of a series of 14 new compounds for use as pH imaging agents using CEST-MRI.^30^ They evaluated the effect of substitution in I45DC scaffold on MRI contrast with the goal of developing compounds with reduced formal charge and osmolarity compared to I45DC-diGlu (**16**) while still maintaining suitable properties for measuring pH values. A number of these compounds were suitable for ratiometric pH measurements within the range of 5.6 to 7.0 including the original I45DC-diGlu (**16**) shown in Figure 4B. The new substituents included amino acids with aliphatic side chains, aromatic side chains, asymmetric substitutions as well as weak electron acceptor groups to influence pH detection ranges. While the aliphatic side chain substitutions led to compounds with suitable labile proton exchange rates, they displayed limited pH detection ranges and reduced quality pH maps due to minimal changes in their saturation transfer signal ratio (ST_ratio_, the contrast metric which is used to construct concentration independent pH maps) over the physiological pH values. Aromatic side chain substitutions resulted in compounds with altered CEST properties, including pH detection ranges of 5.0 to 6.2. Among these, diTrp (**20**) showed promise, but it tended to aggregate at low pH. The study also explored the impact of attaching basic and neutral substituents to the I45DC scaffold, leading to the development of nonionic and cationic I45DC pH sensors. The cationic I45DC-diEda (**19**) exhibited the best overall performance, with a broad pH detection range (4.5 to 7.4) and suitable *k*_ex_ for clinical field strengths.

Toxicity studies on HEK293 cells indicated that I45DC-diEda (**19**) had higher toxicity levels compared to I45DC-diGlu (**16**) and iopamidol, suggesting that the latter was better tolerated by the cells. As a result, I45DC-diGlu was selected for further in vivo testing. In a mouse model of urinary tract obstruction (UTO), the CEST imaging with I45DC-diGlu (**16**) revealed differences in contrast kinetics and pH maps between the unobstructed and obstructed kidneys (Figure 4). The obstructed kidneys displayed an elevated pH. I45DC-diGlu (**16**) demonstrated the ability to measure a wide range of pH values, making it a promising candidate for pH imaging.

#### Free Base Porphyrins and Chlorin

**Figure 5:**
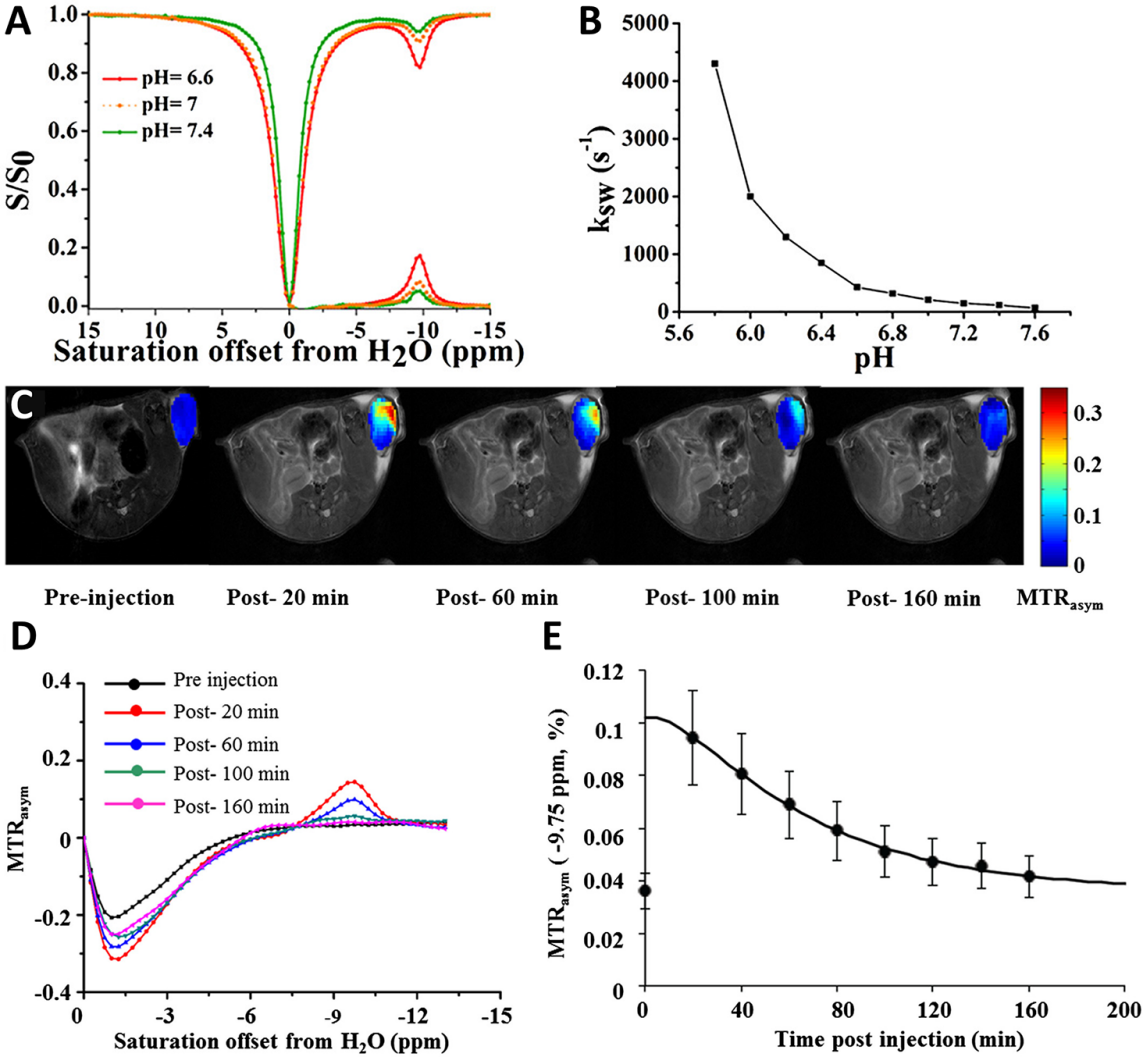
CEST characteristics of TPPS4 at 37 °C. (A) Z-spectrum and magnetization transfer ratio asymmetry (MTRasym) for 12.5 mM TPPS4 (**21**) at pH 6.6, 7.0, and 7.4 using a saturation field strength (ω1) of 5.4 μT. (B) pH dependency of the exchange rate constant (*k*_sw_) based on saturation power data for TPPS4 (**21**). (C) CEST contrast maps at −9.75 ppm preinjection and postinjection at 20, 60, 100, and 160 min of the TPPS4 (**21**). (D) MTRasym for a region of interest (ROI) encompassing the entire tumor with preinjection data (black), 20 min postinjection (red), 60 min postinjection (blue), 100 min postinjection (green), and 160 min postinjection (pink). (E) Temporal evolution of MTRasym (−9.75 ppm) for ROIs covering the entire tumor after intrathecal injection of TPPS4 (**21**) with ω1 = 3.6 μT (*n* = 4). Abbreviations: ω1 - Saturation field strength; TPPS4 - Tetraphenylporphine sulfonate; ROI - Region of Interest. (Adapted with permission from Ref (31), John Wiley and Sons.)

Free-base porphyrins are aromatic macrocycles with a large π electron system and intramolecular hydrogen bonds which allow exceptionally large labile proton chemical shifts.^31^ Zhang et al. investigated a range of free-base porphyrins in 2019, including tetraphenylporphine sulfonate (TPPS4) (**21**), chlorin e6 (**22**), tetrakis (4-carboxyphenyl) porphyrin (TCPP) (**23**), hematoporphyrin (**24**), and uroporphyrin I (**25**). These compounds displayed varying CEST properties, with some showing excellent water solubility and distinct CEST peaks. This study revealed well-defined CEST signal for tetraphenylporphine sulfonate (TPPS4) (**16**) showing an upfield chemical shift of −9.75 ppm from water; thus, isolating the signal for good CEST contrast. The authors further evaluated the properties of TPPS4 (**21**) in vitro, including proton exchange rates and the concentration-dependence of its CEST contrast. Notwithstanding the excellent chemical shift and contrast enhancement, porphyrins can present complications such as metal ligation diminishing CEST contrast, self-aggregation, and limited water solubility. However, these issues were not observed with TPPS4 (**21**), which was also found to be compatible with human serum. In an in vivo study using mice with tumor xenografts, TPPS4 (**21**) was injected intratumorally. CEST imaging before and after injection revealed the distribution of TPPS4 (**21**) within the tumor, with a peak CEST contrast of 9.5% (Figure 5). The unique feature of certain porphyrins and chlorins is their upfield CEST signals, which are well-suited for specific detection due to minimal background signals. This property could open up applications in the field of photomedicine and metabolic disorder detection, providing valuable information for medical diagnoses and therapies.

Tetrakis(*N*-methyl-4-pyridinium)-porphyrin (TmPyP)(**26**) is also emerging as a potential diaCEST MRI contrast agent due to its easily synthesizable nature, high water solubility, and pH-independent contrast efficiency in the physiological range of 6.6 to 8.3. TmPyP, lacking a metal center, exhibits good water solubility at acidic pH levels, essential for excretion, and demonstrates a 15% CEST effect at physiological conditions.^32^

#### Hydrazones and Hydrazides

A study by Dang et al. in 2018 focused on the development of hydrazo-CEST, designed to selectively detect aldehydes using hydrazones within physiological conditions using MRI.^33^ These agents are created by converting the weakly nucleophilic aromatic amine in anthranilic acid into an α-nucleophilic hydrazine. The authors observed that the hydrazine form did not produce any detectable signal using CEST MRI. However, when the hydrazine was converted into a hydrazone, a significant CEST contrast signal could be generated. This property allows for the selective sensing of endogenous reactive carbonyls, making it a valuable tool for detecting aldehydes associated with various cellular processes. The researchers also investigated the structural requirements for hydrazo-CEST, emphasizing the importance of the intramolecular hydrogen bond and the substituents conjugated to the nitrogen with this labile proton. The CEST signal was impacted by the substitution of the phenyl ring, concentration, and pH. Differences in CEST signal were observed for hydrazones derived from aliphatic and aromatic aldehydes and could be attributed to variations in electron density around the ring-proximal nitrogen. The 5-methoxy-substituted analogues (**27**, **28**, **29**) were found to be ideal for endogenous aldehyde detection, and exhibited superior dynamic range for aliphatic aldehydes and enhanced signal generation at neutral pH. This appears to be a promising probe for detection of aldehydes through MRI contrast changes.

Another interesting class of IM-SHY agents are acyl hydrazides, organic molecules containing the (−C(=O)–NHNH_2_) functional group which can convert to acyl hydrazones by condensing with aldehydes or ketones. Acyl hydrazides have gained recognition in drug design and medicinal chemistry due to their versatility and can establish stable intramolecular hydrogen bonding through p-π conjugation. Bo et al. investigated a number of acyl hydrazides in 2023.^34^ Among the investigated acyl hydrazides, benzoyl-substituted acyl hydrazides (**31**) demonstrated strong CEST contrast, while benzenesulfonyl and other substitutions yielded no significant contrast. Aromatic acyl hydrazides with hydrogen bonding potential and electron-withdrawing groups showed enhanced CEST contrast (**32**). Similarly, picolinamide (**33**), exhibited enhanced contrast due to the electron-withdrawing heteroaromatic moiety. The study also extended to aliphatic acyl hydrazides (**35**, **36**, **37**), highlighting that acetylhydrazide exhibited significant CEST contrast, with well-tuned exchange rates. Carbohydrazide, which featured two -NHNH2 groups, displayed CEST contrast, albeit with a smaller chemical shift. Three novel acyl hydrazides (**35**, **36**, 3**7**) were identified with strong CEST contrast and a labile proton resonating >3.5 ppm. Adipic acid dihydrazide (ADH) (**37**) a hydrophilic aldehyde often used as a cross-linking reagent, was identified as particularly suitable and found to maintain properly tuned chemical exchange for CEST up to pH ≤ 6.0. Toxicity studies in 4T1 cells indicated ADH’s (**37**) safety and suitability as a CEST agent. With its small molecular weight and good water solubility, ADH (**37**) emerged as a promising contrast agent for perfusion imaging. In vivo testing of ADH (**37**) in a mouse model of breast cancer demonstrated an increase in CEST MRI contrast in tumor tissue. Furthermore, they showed that ADH (**37**) can be conjugated to a hydrophilic aldehyde which has polyethylene glycol (PEG) chains to form an acyl hydrazone with enhanced water solubility and biocompatibility (**30**). This compound exhibited a robust CEST signal at 6.4 ppm, which is comparable to previously reported hydrazone agents based on phenyl hydrazine. The CEST signal intensity decreased with rising pH, demonstrating the potential of acyl hydrazone as a CEST contrast agent with larger chemical shifts (>6 ppm).

In summary, hydrazo-CEST using acyl hydrazones appear to be a promising class of CEST imaging agents. They benefit from an inherent reduction in background signal because of the isolated labile protons on these agents (∼6.4 ppm from water). This should lead to strong contrast-to-noise ratios and improved imaging signal specificity. Furthermore, these can act as sensors with the “turn-on” mechanism independent of the specific binding of target aldehydes, making it capable of detecting both small molecule aldehydes and those derived from biomacromolecules. As such, these compounds add a new element for using CEST MRI contrast to sense changes in molecular environment.

#### Diacetamide Analogues

Pandey et al. synthesized diacetamide analogues for development as CEST agents in 2022, with the key difference being the position of the NHCOCH_3_ groups on the phenyl ring.^35^ The study explored the comparative CEST efficiency of two isomeric derivatives of diacetamide, both possessing two equivalent exchangeable protons but with differing hydrogen-bonding capabilities. *N*,*N*′-(1,2-phenylene) diacetamide (**38**) had NHCOCH_3_ group substituted at ortho position and exhibited intramolecular hydrogen bonding. The in vitro CEST efficiency of **38** was observed to be 25.4% at Δω ∼ 5 ppm with concentration = 15 mM, saturation B_1_= 5 μT, saturation time = 3 s, magnetic field strength (B_0_) = 9.4 T. These appear to also hold promise for future in vivo studies.

#### Impact of Intramolecular Hydrogen Bonding on CEST Contrast

Yang and colleagues first demonstrated the impact of intramolecular hydrogen bonding on CEST contrast. They also demonstrated “tuning” of CEST contrast via aromatic ring substitution in salicylic acid analogues; modulating p*K*_a_ and chemical shift by steric and electronic effects of the substituents.^10^ Substitutions at position 3 (ortho to IM-SHY core), 6 (ortho to carboxylate anion), positions 4 and 5 (para to carboxylate anion and IM-SHY core, respectively) reveal interesting trends. Most inductive electron-donating and withdrawing groups did not dramatically change the p*K*_a_ at 4- and 5-position substitutions. Electron-donating groups para to the IM-SHY proton (hydroxyl, amino) resulted in lower p*K*_a_ values and chemical shift at 8.5 ppm. Electron-withdrawing groups in para-position and meta inductive substitutions shifted the chemical shift slightly downfield, with lower p*K*_a_ values. Substituents like 3,5-dinitrosalicylic acid failed to give contrast due to predominant deprotonation at neutral pH. The *k*_ex_ values were quite similar to salicylic acid, indicating the buffering effect of the carboxylate anion on moderate p*K*_a_ changes. Substitution at the 6-position resulted in more nuanced behavior. Subtle stereobulky modifications dramatically changed hydrogen bonding between carboxylate and IM-SHY proton. Some substitutions resulted in faster *k*_ex_ values (11–12 times) compared to salicylic acid, making them less suitable for low-field MR applications. 2,6-dihydroxybenzoic acid with two −OH groups exchanged slowly, suggesting the importance of carboxylate solvation in buffering *k*_ex_. 3-Position substitutions gave higher chemical shifts than salicylic acid with tunable *k*_ex_. Increasing the size of the substituent at the 3-position slowed down *k*_ex_ due to limited water access.

In 2016, Yang and colleagues demonstrated how the hydrogen bonding properties could be tuned for imidazoles based on the position and number of Ns in the imidazole ring and the ring substitution.^29^ They found that the presence of carbons between the nitrogens in imidazoles played a critical role for balancing the p*K*_a_ of the exchangeable proton and the hydrogen bonding. The additional hydrogen bonds produced by having substituents at the 4 and 5 positions were also found to stabilize the folded conformation.

Pandey et al. further demonstrated the impact of hydrogen bonding on CEST contrast efficiency using acetamides in 2022.^35^ As hydrogen bonding affects lability of the exchanging proton group, it is believed that hydrogen bonding directly influences CEST efficiency. By comparing two diacetamide analogues with similar molecular weight (one with intramolecular hydrogen bonding and another with intermolecular hydrogen bonding network), they made some very important observations. The intraMHB (intramolecular hydrogen bonding) analogue was found to have better solubility. With increase in temperature slightly above body temperature, the hydrogen bonding network weakens and the CEST efficiency drops rapidly.

## Future Outlook: Applications and Potential Clinical Relevance

CEST MRI is a powerful imaging method and has proven highly useful in various medical applications. From examining brain tumors,^36^ especially gliomas, it is now in trials to help assess a number of tumor types including breast tumors, pelvic tumors, digestive tumors, and lung tumors.^37^ CEST MRI is also being used to monitor stroke progression, study neurodegenerative diseases, and assess musculoskeletal disorders.^38^ CEST MRI is also emerging as a promising tool for studying kidney diseases, offering a noninvasive way to detect and understand the progression of kidney disease.^14^ Studies have delved into its application in chronic kidney disease (CKD), aiming to understand tissue composition changes and molecular alterations associated with different CKD stages. CEST MRI has proven useful in differentiating between benign and malignant renal tumors, providing valuable insights into the molecular features of these lesions.^37^ It has been explored in assessing renal fibrosis, ischemia-reperfusion injury, and diabetic nephropathy, offering a means to study molecular changes associated with these conditions.^39^ CEST MRI has been applied to investigate renal inflammation, aiding in the identification of specific biomarkers indicative of inflammatory processes in the kidneys.^40^ It is expected that CEST MRI can expand the diagnostic potential of MRI significantly.

The application of pH mapping through CEST MRI offers a noninvasive means to assess tissue acidity levels. For cancer staging and treatment monitoring, identifying acidic regions within tumors may allow improved biopsy sampling of tissue and improve the evaluation of therapeutic responses. For ischemic strokes, CEST MRI allows the assessment of pH changes in affected brain regions, contributing to stroke evaluation and management. Inflammatory diseases, renal disorders, neurological conditions, and cardiovascular issues also could benefit from pH mapping’s ability to noninvasively monitor tissue acidity. Additionally, pH mapping finds utility in drug delivery monitoring, wound healing assessment, and holds promise for optimizing drug formulations. As this field advances, integrating pH mapping into routine clinical imaging protocols could significantly enhance diagnostic precision and patient care across various medical disciplines.

Intramolecular hydrogen bonding-based CEST MRI contrast agents represent an innovative approach to maximize the sensitivity and specificity of CEST MRI contrast agent detection and maximize the performance of CEST MRI pH mapping for assessing tissue acidity levels. These contrast agents are designed with specific molecular structures that allow intramolecular hydrogen bonds to form to tune the exchange properties for maximal performance and also shift labile protons far away from most endogenous signals. As the local pH environment varies, the exchangeable protons involved in intramolecular hydrogen bonding will undergo alterations in their chemical exchange rates, enabling CEST MRI to detect and quantify these changes. This mechanism provides a more direct and targeted means of measuring pH compared to traditional CEST agents.

To broaden the clinical application of CEST MRI agents, recent efforts have prioritized biocompatibility, water solubility, and suitable chemical exchange properties under physiologically relevant conditions, etc. For example, compounds with amine functionalities, such as acyl hydrazides and hydrazones display pH-dependent contrast which is readily detected within physiologically relevant ranges. Additionally, the use of hydrophilic polymers, as seen in polymeric salicylate probes, ensure stability and facilitate targeted imaging. These strategies enhance the translational potential of diaCEST imaging in clinical settings. All probes highlighted in Figure 2 (except **20**) exhibit high levels of CEST contrasts in physiologically relevant pH range (7.1–7.6). As the contrast is pH dependent, performance of these agents at acidic pH (6.5–6.9) is beneficial for studying tumors and organs with slightly acidic tissue such as the kidney.

Intramolecular hydrogen bonding-based CEST MRI contrast agents are at their infancy although there have been a number of research and preclinical studies conducted which have been described above to explore their potential. The advantages of intramolecular hydrogen bonding-based CEST MRI contrast agents include improved sensitivity, specificity and tuning of the pH sensitivity of the CEST signals, which will result in increased spatial resolution, better discrimination of agent from background. They also offer a more selective response to pH changes, enhancing the precision of pH mapping in biological tissues. The ability to design contrast agents with tailored properties allows for a customizable approach to meet the specific requirements of different imaging scenarios. In a murine model with unilateral urinary tract obstruction, Bo et al. demonstrated pH mapping using I45DC-diGlu (**16**) in 2022 and generated concentration independent pH maps using CEST MRI. A noticeable difference in contrast uptake was observed between the obstructed and unobstructed kidneys, with the unobstructed kidney exhibiting a pH of approximately 6.5. In contrast, the obstructed kidney displayed an elevated pH, accompanied by a broader range of pH values. These findings suggest that the I45DCs exhibit impressive imaging characteristics, showcasing their potential for a variety of medical imaging contexts, especially for renal imaging.

While intramolecular hydrogen bonding-based CEST MRI contrast agents show great promise, it is crucial to acknowledge certain limitations associated with CEST MRI as a whole. One notable challenge is the susceptibility to confounding factors such as magnetic field inhomogeneity and transmit B_1_ field inhomogeneity, which can impact the accuracy of pH measurements. Additionally, the dependence on various parameters, including exchange rate, offset frequencies, and probe concentrations, introduces complexities that must be carefully addressed for reproducible results and similarity across different instruments. Furthermore, the translation of preclinical successes to clinical applications requires thorough validation and standardization. Another important consideration is the effect of applied magnetic field on the contrast enhancement. For example, 2,5-dihydroxyterephthalic acid (8) showed a contrast of 17.1% at 7.4 T which decreased to 9% at 3 T for a concentration of 10 mM.^10^ Despite these challenges, ongoing research endeavors aim to overcome these limitations, emphasizing the importance of continued innovation and refinement of IM-SHY agents and CEST MRI as a whole.

## Summary

The emergence of intramolecular hydrogen bonding-based CEST MRI contrast agents represents a cutting-edge design strategy that holds great promise in the field of molecular imaging and pH mapping. The innovative approach offers enhanced sensitivity and specificity in assessing tissue acidity levels, addressing key limitations associated with traditional metal-based MRI contrast agents. The advantages of improved pH sensitivity increased spatial resolution, and a customizable design underscore the potential of intramolecular hydrogen bonding-based CEST MRI contrast agents for diverse clinical applications.

Preclinical studies demonstrate the impressive imaging characteristics and potential clinical applications, particularly in renal imaging. The observed differences in contrast uptake between obstructed and unobstructed kidneys and concentration-independent pH maps, showcase the clinical relevance of these agents. Beyond renal imaging, IM-SHY agents hold promise in oncology for characterizing tumor acidity and monitoring treatment responses, as well as in neuroimaging for studying pH variations in the brain.

As this innovative approach continues to evolve, ongoing research aims to expand the role of intramolecular hydrogen bonding-based CEST MRI contrast agents to more applications. The customizable nature of these agents opens avenues for tailoring responses to specific clinical contexts, further enhancing diagnostic precision. The future outlook for intramolecular hydrogen bonding-based CEST MRI contrast agents is optimiztic, with the potential to revolutionize molecular imaging, provide valuable insights into physiological and pathological conditions, and contribute significantly to advancing clinical diagnostic capabilities.
